# Role for Lipids Secreted by Irradiated Peripheral Blood Mononuclear Cells in Inflammatory Resolution in Vitro

**DOI:** 10.3390/ijms21134694

**Published:** 2020-06-30

**Authors:** Layla Panahipour, Evgeniya Kochergina, Maria Laggner, Matthias Zimmermann, Michael Mildner, Hendrik J. Ankersmit, Reinhard Gruber

**Affiliations:** 1 Department of Oral Biology, Medical University of Vienna, Sensengasse 2a, 1090 Vienna, Austria; layla.panahipour@meduniwien.ac.at (L.P.); eugeniya20@mail.ru (E.K.); 2 Laboratory for Cardiac and Thoracic Diagnosis, Regeneration and Applied Immunology, Währingergürtel 18-20, 1090 Vienna, Austria; maria.laggner@meduniwien.ac.at (M.L.); hendrik.ankersmit@meduniwien.ac.at (H.J.A.); 3 Division of Thoracic Surgery, Medical University of Vienna, Währingergürtel 18-20, 1090 Vienna, Austria; 4 Department of Oral and Maxillofacial Surgery, Medical University of Vienna, Währingergürtel 18-20, 1090 Vienna, Austria; matthias.zimmermann@meduniwien.ac.at; 5 Research Division of Biology and Pathobiology of the Skin, Department of Dermatology, Medical University of Vienna, Währingergürtel 18-20, 1090 Vienna, Austria; michael.mildner@meduniwien.ac.at; 6 Department of Periodontology, School of Dental Medicine, University of Bern, Freiburgstrasse 7, 3010 Bern, Switzerland; 7 Austrian Cluster for Tissue Regeneration, Donaueschingenstraße 13, 1200 Vienna, Austria

**Keywords:** wound healing, secretome, inflammation, macrophages, apoptosis, necroptosis, periodontitis

## Abstract

Periodontal inflammation is associated with dying cells that potentially release metabolites helping to promote inflammatory resolution. We had shown earlier that the secretome of irradiated, dying peripheral blood mononuclear cells support in vitro angiogenesis. However, the ability of the secretome to promote inflammatory resolution remains unknown. Here, we determined the expression changes of inflammatory cytokines in murine bone marrow macrophages, RAW264.7 cells, and gingival fibroblasts exposed to the secretome obtained from γ-irradiated peripheral blood mononuclear cells in vitro by RT-PCR and immunoassays. Nuclear translocation of p65 was detected by immunofluorescence staining. Phosphorylation of p65 and degradation of IκB was determined by Western blot. The secretome of irradiated peripheral blood mononuclear cells significantly decreased the expression of IL1 and IL6 in primary macrophages and RAW264.7 cells when exposed to LPS or saliva, and of IL1, IL6, and IL8 in gingival fibroblasts when exposed to IL-1β and TNFα. These changes were associated with decreased phosphorylation and nuclear translocation of p65 but not degradation of IκB in macrophages. We also show that the lipid fraction of the secretome lowered the inflammatory response of macrophages exposed to the inflammatory cues. These results demonstrate that the secretome of irradiated peripheral blood mononuclear cells can lower an in vitro simulated inflammatory response, supporting the overall concept that the secretome of dying cells promotes inflammatory resolution.

## 1. Introduction

The integrity of the tooth-supporting periodontium and the oral mucosa serving as a barrier shielding the oral tissues against the microflora of the oral cavity requires continued renewal [1,2]. This renewal is achieved by the controlled dying of old or damaged cells and the concomitated replacement by new cells, a process termed homeostasis [3], a process that might be reflected by systemic levels of lipid metabolites malondialdehyde acetaldehyde [4], hormones such as vitamin D [5], and circulating cells including those of the endothelial linage [6]. Apoptosis and senescence are the most important mechanisms to eliminate damaged cells [7,8] and the role of autophagy in the pathogenesis of periodontal disease is increasingly recognized [9]. If this homeostasis is disturbed, for example in response to chronic inflammation, catabolic changes culminate in tissue atrophy as observed at sites of chronic periodontitis [10] and periimplantitis [11]. Similarly, catabolic changes are also observed at sites of severe oral mucositis mainly caused by radiation and chemotherapy in oncology [12]. One fundamental principle to maintain homeostasis is that the dying cells release metabolites as ‘good-bye’ signals that help to maintain tissue integrity [13].

Apoptosis is a programmed cell death that culminates in the activation of caspase-3 by cleavage of the pro-enzyme and DNA fragmentation, the latter being detected by TdT-mediated dUTP-biotin nick end labeling (TUNEL) staining. In human gingivitis and periodontitis, caspase-3 was expressed mainly in the cells of lamina propria and to a lesser extent in the sulcular epithelium. In periodontitis, TUNEL-positive cells were present in oral, sulcular, and junctional areas with the majority in the lamina propria beneath the junctional epithelium [14]. Also, caspase-3 and TUNEL-positive cells were detected in rat periodontitis models [15,16]. Others reported that LPS increased the percentage of apoptotic and necrotic cells in gingival tissues of IDO-KO mice [17]. Importantly, CD68-positive macrophages show cleaved caspase-3 in periodontitis and gingivitis patients [14]. Thus, apoptotic cells of the macrophage lineage are present in the inflamed periodontium. The question arises if the metabolites released by dying cells may dampen inflammation and may help to regain tissue homeostasis. 

Though pre-clinical studies transplanting stem cells for cardiac repair generated promising results in regenerative medicine [18], pioneer clinical trials failed to reproduce any of the encouraging results [19]. More recent studies provided potential explanations for the observed discrepancies. First, Togel and colleagues reported that tissue integration of transplanted cells was poor [20]. In addition, administration of conditioned medium from mesenchymal stem cells after cardiac injury revealed that secreted factors, rather than cells themselves, exert beneficial paracrine effects and predominantly account for the promising initial findings [21,22]. These results provided the basis for a novel approach in regenerative medicine, where application of cell-derived secretomes and conditioned cell culture media were proposed. 

In 2009, Ankersmit et al. demonstrated that infusion of irradiated, dying peripheral blood mononuclear cells (PBMCs) effectively promoted regeneration of the infarcted myocardium [23] and regenerative effects of the secretome obtained from PBMCs in acute myocardial infarction [24]. In contrast to stem cells, PBMCs represent an easily accessible and rich source for cellular secretomes and comparable action spectra of progenitor cells and PBMCs have been demonstrated [25]. Support for an active contribution of dying cells to support tissue homeostasis and regeneration come from observations that metabolites from apoptotic lymphocytes and macrophages attenuate arthritic symptoms in preclinical studies [13]. Our translational approach is based on the use of the secretome of human PBMCs driven into ex vivo radiation-induced apoptosis and necroptotic cell death [26]. Necroptosis was identified as programmed necrosis [27]. It was found that the monocytes fraction predominantly accounts for the pro-angiogenic activity of the secretome [26]. Evidence for its efficacy to support tissue regeneration comes from preclinical observations showing that the secretome of PBMCs exerts beneficial effects in various indications, including myocarditis [28], chronic heart failure [29], spinal cord injury [30], stroke [31], and wound healing [32]. The secretome of irradiated PBMCs has already been shown to promote angiogenesis [26], however, its role in modulating an inflammatory response and resolution of inflammation has not been investigated so far. Lipids are known, potent modulators of the immune system, and presence of (non-)oxidized lipids and resolvins in the secretome of irradiated PBMCs has been reported previously [33,34]. Though immunomodulatory actions of lipids have been demonstrated in dendritic cell-mediated inflammation [34], the effects of lipids secreted by irradiated PBMCs on macrophages have not been elucidated so far.

From a regulatory point of view, cell-derived secretomes are classified as biological medicinal products. Prior to application in humans, several safety and quality aspects, such as viral clearance and a controlled manufacturing process, have to be considered. Previously, efficacy of viral clearance has been demonstrated by Gugerell et al. [35]. Moreover, toxicological testing with subcutaneous and intravenous application of PBMC secretome has been performed with no adverse events related to the PBMC secretome [36]. Reproducibility and good manufacturing practice-compliant production of the PBMC secretome have been successfully demonstrated [37] and a phase I clinical trial confirmed pre-clinical findings when no secretome-related adverse events were observed when topically administering autologous irradiated PBMC-derived secretomes in healthy volunteers (ClinicalTrials.gov Identifier: NCT02284360) [38]. These works provide the basis for the regulatory approval of a clinical trial phase II applying PBMC secretomes to promote wound closure of diabetic foot ulcers (EudraCT number 2018-001,653-27) and future clinical use of cell-derived secretomes in regenerative medicine.

The rationale of the study is based on the concept that macrophages undergo apoptosis in periodontitis and gingivitis patients [14] and we use the secretome of irradiated necroptotic and apoptotic PBMCs to simulate this situation based on a proof-of-concept research approach. Here, we took advantage of established in vitro models with murine bone marrow macrophages and the respective RAW264.7 cell line, as well as gingival fibroblasts, where an inflammatory response can be induced by exposure to LPS or saliva, and the pro-inflammatory cytokines IL-1β and TNFα, respectively. The possible decline of an inflammatory response by the secretome is determined based on a panel of in vitro assays. Furthermore, we sought to determine whether lipids secreted by irradiated PBMCs exert anti-inflammatory effects. We present here that the secretome of PBMCs that were subjected to ex vivo radiation exerts a profound inhibition of the inflammatory response in macrophages and oral fibroblasts and that lipids predominantly mediate the observed effects.

## 2. Results

### 2.1. Secretome Suppresses Inflammation in Primary Macrophages and RAW264.7 Cells

**Figure 1:**
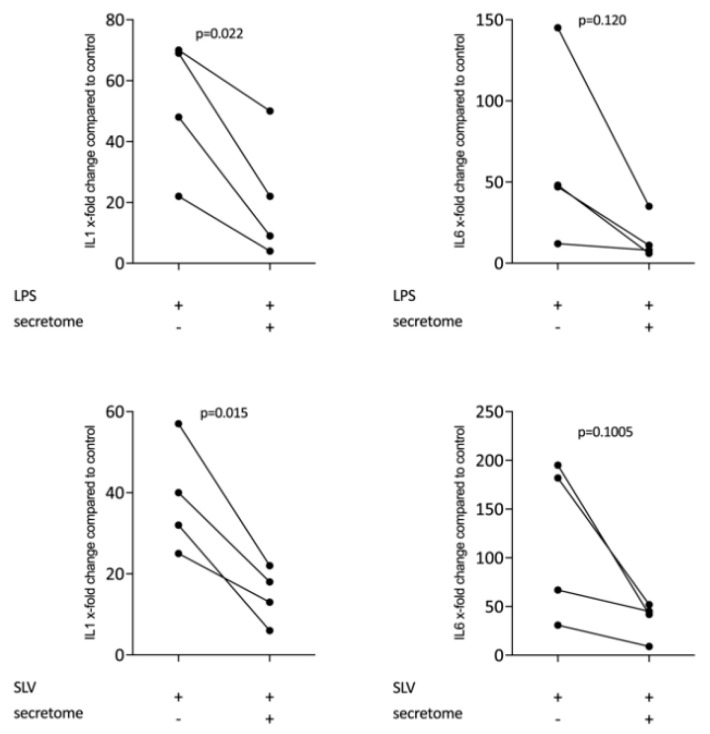
Secretome of irradiated peripheral blood mononuclear cells (PBMCs) suppresses inflammation in primary macrophages. Murine bone marrow-derived macrophages were exposed to secretome corresponding to 1 × 10^7^ irradiated PBMCs/mL. Inflammation was provoked by exposure to LPS 100 ng/mL or 5% Saliva (SLV). X-fold change of expression compared to control is indicated. Dot-blots represent independent experiments. *p*-values were calculated by paired t-test.

**Figure 2:**
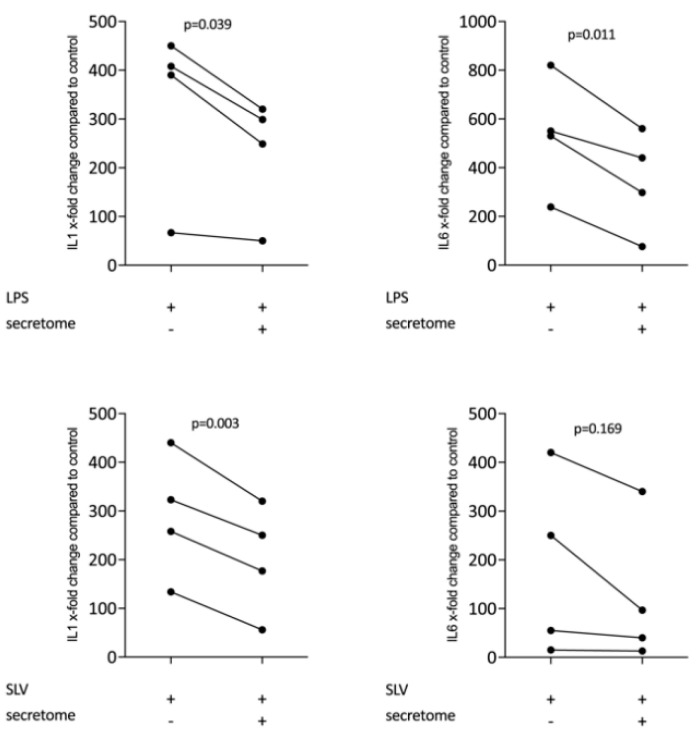
Secretome of irradiated PBMCs suppresses inflammation in RAW264.7 cells. RAW264.7 cells were exposed to secretome corresponding to 1 × 10^7^ irradiated PBMCs/mL. Inflammation was provoked by LPS 100 ng/mL or 5% Saliva (SLV). X-fold change of expression compared to control is indicated. Dot-blots represent independent experiments. *p*-values were calculated by paired t-test.

**Figure 3:**
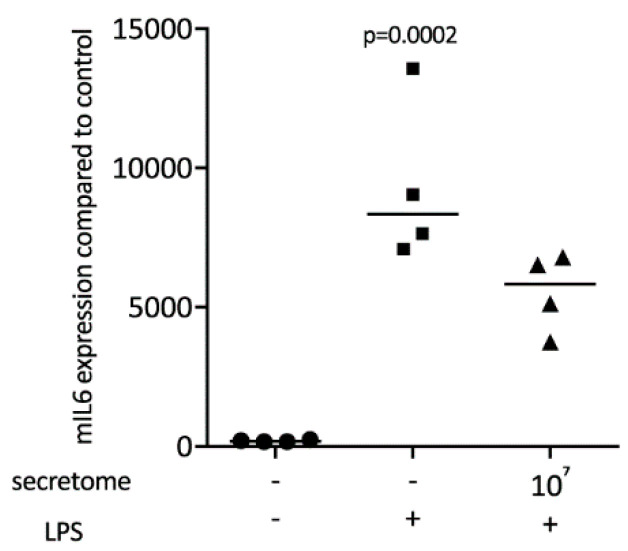
Secretome of irradiated PBMCs suppresses IL6 release in primary macrophages. IL6 protein levels in the supernatant were determined by immunoassay. Murine bone marrow-derived macrophages were exposed to secretome. The inflammation was provoked by LPS 100 ng/mL. Dot-blots represent independent experiments. *p*-values were calculated by Kruskal–Wallis test.

We have shown previously that the secretome of γ-irradiated PBMCs, and in particular the monocyte population, holds a pro-angiogenetic activity as determined in an aortic ring assay [26]. To determine whether this secretome can also promote inflammatory resolution, we have now exposed primary macrophages and RAW264.7 cells to the secretome of γ-irradiated PBMCs and activated TLR-signaling with LPS or saliva to provoke the expression of pro-inflammatory cytokines [39]. We report here that the secretome of γ-irradiated PBMCs corresponding to 1 × 10^7^ cells/mL substantially decreased the expression of IL1 and IL6 in primary macrophages (Figure 1 and Figure S1) and RAW264.7 cells (Figure 2), equally effective after LPS or saliva treatment. The decrease in IL6 production by primary macrophages was further corroborated on the protein level by immunoassay (Figure 3). The secretome also exerts it anti-inflammatory activity expressed as in macrophages from Nrf2 knockout mice. The secretome of γ-irradiated PBMCs decreases LPS or saliva-induced IL1 expression in primary macrophages from wildtype but also from Nrf2 knockout mice (Table 1).

### 2.2. Secretome Hinders p65 Phosphorylation and Nuclear Translocation

**Figure 4:**
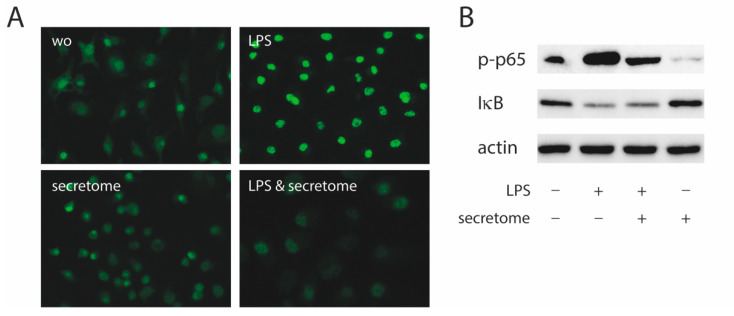
Secretome hampers p65 nuclear translocation and phosphorylation. Murine bone marrow-derived macrophages were exposed to secretome followed by adding LPS for 25 min. (**A**) The p65 nuclear signal in macrophages being exposed to LPS shows a reduced intensity when combined with the secretome. (**B**) The secretome reduces LPS-induced phosphorylation of p65 in primary macrophages. Serum-starved murine bone marrow-derived macrophages were exposed to secretome and LPS for 25 min. Targets were detected by antibodies raised against phosphor p65 and IκB.

To identify the underlying molecular mechanisms by which the secretome exerts its anti-inflammatory activity, the p65 nuclear translocation in primary macrophages exposed to LPS was investigated (Figure 4A). Consistent with our findings, the LPS-induced nuclear translocation of p65 in primary macrophages was remarkably lower in the presence of the secretome (Figure 4A). The secretome attenuated the phosphorylation of p65 induced by LPS in primary macrophages (Figure 4B). The secretome had no impact on the degradation of IκB in primary macrophages when exposed to LPS (Figure 4B).

### 2.3. Secretome Suppresses Inflammation in Primary Gingival Fibroblasts

**Figure 5:**
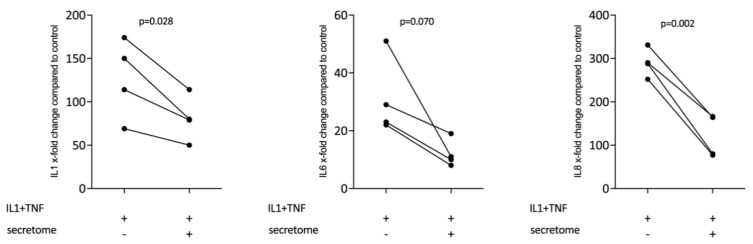
Secretome of irradiated PBMCs suppresses inflammation in primary gingival fibroblasts. Gingival fibroblasts were exposed to secretome corresponding to 1 × 10^7^ irradiated PBMCs/mL. The Inflammation was provoked by IL-1β and TNFα (both at 10 ng/mL). X-fold change of expression compared to control is indicated. Dot-blots represent independent experiments. *p*-values are based on a paired t-test.

We next determined whether the decreased expression of inflammatory cytokines is restricted to myeloid cells or also accounts true for cells of the mesenchymal lineage. We therefore assessed whether the presence of the secretome from γ-irradiated PBMCs affected the IL-1β and TNFα-induced expression of inflammatory cytokines in gingival fibroblasts. Consistent with this assumption, the secretome corresponding to at 1 × 10^7^ PBMCs/mL significantly reduced the expression of IL1, IL6, and IL8 in primary gingival fibroblasts (Figure 5).

### 2.4. Secretome Lipid Extracts Suppress Inflammation in Macrophages

**Figure 6:**
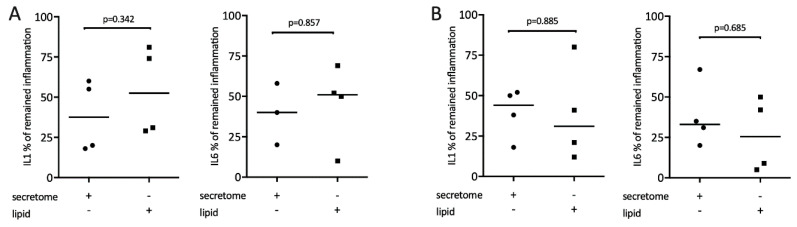
Lipid extracts of irradiated PBMCs suppress inflammation in primary macrophages. Murine bone marrow-derived macrophages were exposed to 1 × 10^7^ secretome and equivalent lipids. Inflammation was induced by exposure to 100 ng/mL LPS (**A**) or 5% saliva (**B**). Expression of inflammatory genes is indicated in percentage (%) compared to stimulated controls (100%). Dot-blots represent independent experiments. *p*-values are based on a Mann–Whitney test.

It is known that lipids exert potent immunomodulatory actions [34]. Therefore, we determine whether the lipid fraction of the secretome from γ-irradiated PBMCs is involved in mediating the reduced expression of inflammatory cytokines. To this end, lipid fractions and secretome were tested in macrophages exposed to LPS or saliva, respectively. Gene expression analysis showed that lipid fractions, similar to the whole secretome, reduced the LPS or saliva-stimulated expression of IL1 and IL6 in macrophages (Figure 6).

## 3. Discussion

Prompted by the evidence that the secretome of dying PBMCs elicits a beneficial effect in pathological conditions linked with inflammation and tissue damage such as myocarditis [28], chronic heart failure [29], spinal cord injury [30], stroke [31], and wound healing [32], and that inflammation-related catabolic changes of the periodontium are associated with the increasing presence of apoptotic macrophages [14], the goal of the work presented here was to examine whether the secretome of irradiated PBMCs can attenuate the expression of inflammatory cytokines. To accomplish this goal, we exposed cells of the macrophage and mesenchymal lineage to the respective secretome while provoking an inflammatory response. The main finding of this work is that the secretome of irradiated PBMCs caused a robust attenuation of the expression of inflammatory cytokines in macrophages and gingival fibroblasts, and that this anti-inflammatory activity is exerted by the lipid fraction of the secretome.

These findings reveal for the first time that, in addition to its potent effects on angiogenesis [26], the secretome of irradiated, dying PBMCs holds a paracrine activity to decrease the NFkB-mediated canonical inflammatory response of macrophages. This previously unrecognized effect of the secretome is not restricted to reduced TLR signaling; also, the IL-1β and TNFα-induced inflammatory response of gingival fibroblasts is attenuated by the secretome. In contrast to our original assumption [40], the anti-inflammatory effect of the secretome is Nrf2-independent based on findings with macrophages from the respective knockout models. Even though no in vitro results with the secretome of PBMCs to modulate an inflammatory response in vitro are available, our data are in support of earlier studies showing that the secretome of mesenchymal cells has an anti-inflammatory activity in vitro. For example, hypoxic periodontal ligament fibroblasts can lower TNFα, COX-2, and iNOS in scratch-injured neurons in vitro [41] and the secretome of adipose tissue fibroblasts can lower inflammatory cytokines in monocytes in vitro [42]. The obvious question arises on what is the active component that mediates the anti-inflammatory activity of the secretome of dying PBMCs.

We have previously shown that irradiated PBMCs differentially expressed genes that encode secreted proteins including those involved in the generation of oxidized phospholipids, particularly oxidized phosphatidylcholines with a known inflammation-modulating property [33]. Secreted lipids, however, had no effects on migration of fibroblasts in a scratch assay [33]. In general, oxidation of phospholipids provides a negative feedback preventing damage to host tissues due to uncontrolled inflammation and oxidative stress [43]. Lipid mediators including those of the cyclopentenone-family exert anti-inflammatory activity through inhibition of NFκB-induced proinflammatory gene expression in RAW264.7 cells [44] and primary macrophages [45,46]. Oxidized low-density lipoproteins were also detected in gingival crevicular fluid from dental patients while their involvement in maintaining tissue homeostasis is unclear [47]. What remains now to be done is to identify which lipids account responsible for the anti-inflammatory activity of the secretome.

The secretome and presumably also the lipid fraction greatly reduced p65 phosphorylation and nuclear translocation. Considering that NF-κB activation requires the phosphorylation, ubiquitination, and subsequent degradation of its inhibitory subunit, IκBα, the secretome failed to block this process. Thus, inhibiting IκBα degradation is not responsible for the lowering of p65 phosphorylation and nuclear translocation by the secretome [29,30]. The data are nevertheless consistent with observations that oxidized low-density lipoprotein (oxLDL) reduces the expression of proinflammatory genes in macrophages [48]. However, in contrast to our observations, this phenomenon was partially dependent on NRF2 [48] and lipoproteins reduced the binding of p65 to the IL6 and CCL5 promoters [48]. Thus, even though the secretome consistently reduced p65 phosphorylation and nuclear translocation in macrophages, the detailed signaling mechanism remains to be identified.

The clinical relevance of our findings remains a matter of speculation. It can be assumed that the secretome of apoptotic CD68-positive macrophages in periodontitis and gingivitis patients may exert an anti-inflammatory activity [14] that is maybe not restricted to the macrophage lineage [14,15,16,17]. Moreover, the secretome of PBMCs that was subjected to ex vivo radiation, at least on the molecular level, represent the natural process of necroptosis though mixed lineage kinase domain-like protein (MLKL) and receptor-interacting protein serine-threonine kinases-3 (RIPK3) [26,27]. Nevertheless, the role of necroptosis in the periodontal homeostasis is increasingly recognized [49]. In vivo, elevated levels of the necroptosis markers, in particular RIPK1 and MLKL, were observed in gingival tissues collected from patients with untreated chronic periodontitis [50]; but so far, the periodontal ligament cells are supposed to undergo necroptosis [50]. Maybe the clinical relevance of the secretome of PBMCs is related to the local application, particularly on periodontitis and periimplantitis, as reported for wound healing [32]. In this case, the secretome might, at least in theory, support the healing of the local periodontal and periimplant defects. Future research should therefore focus on how periodontal pathogens directly affect the process of necroptosis, not only in macrophages, but also in cells of the connective tissue and the epithelial barrier – and if the cellular response involves the release of lipids that defend the inflammatory reaction; for example oxLDL can be detected in the gingival crevicular fluid from dental patients that might be associated with virulence factors released by the pathogenic microbiota [47] and oxLDL reducing the expression of proinflammatory genes in macrophages [48]. If oral pathogens control the production of lipids other than prostanoids by cells of the oral cavity has not been identified but for example, macrophages can release sphingolipids [51]. However, it is the oral pathogens including P. gingivalis producing lipids such as sphingolipids [52], serine-glycine dipeptide lipid [53] and short-chain fatty acids [54] that can in turn manipulate the host inflammatory response in periodontitis and other inflammatory diseases.

In conclusion, the main finding of the present study was that the secretome of irradiated PBMCs holds a strong anti-inflammatory activity in macrophages and gingival fibroblasts being associated with decreased phosphorylation and nuclear translocation of p65. Even more intriguingly, the anti-inflammatory effects of the lipid fraction open the door for future investigations that goes beyond dental research.

## 4. Materials and Methods

### 4.1. Bone Marrow-Derived Macrophages, RAW264.7 Cells and Gingival Fibroblasts

BALB/c mice at the age of 6–8 weeks were purchased from Animal Research Laboratories, Himberg, Austria. The femora and tibiae of the mice were removed after scarifying and bone marrow cells were collected. Bone marrow cells were seeded at 1 × 10^5^ cells/cm^2^ into 12-well plates and grown for 5–7 days in Dulbecco’s Modified Eagle Medium (DMEM) supplemented with 10% fetal calf serum, 1% of 10,000 units penicillin and 10 mg streptomycin/mL (Sigma, St Louis, MO, USA) and with 20ng/mL mouse macrophage colony-stimulating factor (M-CSF; ProSpec-Tany TechnoGene Ltd., Rehovot, Israel). RAW 264.7 macrophage-like cells (ATCC; LGC Standards GmbH, Wesel, Germany), expanded in regular DMEM growth medium without supplement. Macrophages were also prepared from Nrf2 knockout and wildtype mice that were kindly provided by T. Kensler, Johns Hopkins University, Baltimore, MD, USA, and M. Yamamoto, University of Tsukuba, Tennoudai, Japan. Human gingival fibroblasts were prepared by explant cultures after approval of the Ethical Committee of the Medical University of Vienna (EK Nr. 631/2007). All cells were cultured under standard conditions at 37 °C, 5% CO_2_, and 95% humidity.

### 4.2. Secretome of PBMCs

Heparinized blood samples for PBMC isolation were obtained from healthy volunteers at the Austrian Red Cross Blood Transfusion Service of Upper Austria, Linz, Austria. All donors provided informed written consent. Briefly, PBMCs were isolated from heparinized blood using density gradient centrifugation via Ficoll-Paque PLUS (GE Healthcare Bio-Sciences AB, Uppsala, Sweden). The buffy coat containing the PBMCs was resuspended at 2.5 × 10^7^ cells/mL in CellGro serum-free medium (CellGenix, Freiburg, Germany), irradiated with Cesium-137 (60 Gy), and cultivated for 24 h [55]. Supernatants were collected by centrifugation at 400× *g* for 9 min and passed through 0.2 µm filters. Methylene blue treatment for viral clearance was performed as described previously [56]. Secretomes were lyophilized and terminally sterilized by high-dose ϒ-irradiation (Gammatron 1500, UKEM 60Co irradiator). Reconstitution was performed by serum-free media to receive a stock concentration equivalent to 2 × 10^7^ cells/mL that was further diluted to a working concentration equivalent to or below 1 × 10^7^ cells/mL. The lipid fraction was prepared based on a traditional chloroform/methanol extraction method [57] with minor modifications [55]. All experiments were performed with batches A000918399132 and A000918399129.

### 4.3. Saliva Preparation

Whole human saliva was collected from the authors (L.P., R.G.) who are non-smokers and gave their informed consent. Saliva flow was stimulated by chewing paraffin wax (Ivoclar Vivadent AG, Schaan, Liechtenstein) without eating and drinking for 1 h prior to collection. Immediately after collection, saliva was centrifuged at 4000× *g* for 5 min. The saliva supernatant was passed through a filter with a pore diameter of 0.2 µm (Diafil PS, DIA-Nielsen GmbH, Düren, Germany). The saliva from the two donors was pooled and frozen stocks were used to provoke the expression of inflammatory cytokines in macrophages [39].

### 4.4. Cell Stimulation

Primary macrophages and RAW264.7 cells were exposed to the secretome and stimulated with 5% saliva or 100 ng/mL LPS for 24 h. Gingival fibroblasts were exposed to the secretome corresponding to 1 × 10^7^ cells/mL in the presence of IL-1β and TNFα (both at 10 ng/mL, ProSpec-Tany TechnoGene Ltd., Rehovot, Israel) in growth medium. After 24 h, gene expression analysis was performed and the supernatant was collected for immunoassays.

### 4.5. RT-PCR and Immunoassay

Total RNA was isolated with the ExtractMe total RNA kit (Blirt S.A., Gdańsk, Poland). Reverse transcription was performed with SensiFAST cDNA kit (Bioline, London, UK). Polymerase chain reaction was done with the SensiFAST master mix (Bioline). Amplification was monitored on the CFX Connect™ Real-Time PCR Detection System (Bio-Rad Laboratories, CA, USA). Primer sequences are provided in Table 2. The mRNA levels were calculated by normalization to the housekeeping gene GAPDH and actin using the ΔΔCt method. For the immunoassay, the mouse IL6 kit was used (R&D Systems, Minneapolis, MN, USA).

### 4.6. Western Blot

Primary macrophages were serum-starved overnight and then preincubated for 10 min with secretome corresponding to 1 × 10^7^ cells/mL before being exposed for 25 min to LPS (100 ng/mL). Cell extracts containing SDS buffer and protease inhibitors (PhosSTOP with cOmplete; Sigma, St. Louis, MO, USA) were separated by SDS-PAGE and transferred onto nitrocellulose membranes (Whatman, GE Healthcare, General Electric Company, Fairfield, CT, USA). Membranes were blocked and the binding of the first antibody raised against phospho- NF-κB p65 and IκB (#3033 and #9242; Cell Signaling Technology, Danvers, MA, USA) and beta-actin (Santa Cruz Biotechnology, Santa Cruz, CA, USA) was detected with the appropriate secondary antibody linked to a peroxidase. Chemiluminescence signals were visualized with the ChemiDoc imaging system (Bio-Rad Laboratories, Inc., Hercules, CA, USA).

### 4.7. Immunofluorescence

To confirm the ability of the secretome to modulate the inflammatory response of primary macrophages to LPS exposure, p65 staining was performed. The immunofluorescent analysis of p65 was performed in primary macrophages plated onto Millicell^®^ EZ slides (Merck KGaA, Darmstadt, Germany) at 15,000 cells/cm^2^. Serum-starved cells were exposed to the secretome for 10 min following inflammatory response provoked by LPS for 25 min. The cells were fixed with 4% paraformaldehyde, blocked with 1% bovine serum albumin and permeabilized with 0.3% Triton. We used rabbit anti-human NFκB p65 (#8242; Cell Signaling Technology, Cambridge, UK) at 4 °C overnight. Detection was performed with the goat anti-rabbit Alexa 488 secondary antibody (CS-4412, Cell Signaling Technology). Images were captured under a fluorescent microscope with a single filter block 455 nm (Oxion fluorescence, Euromex, Arnheim, Netherlands).

### 4.8. Statistical Analysis

All experiments were repeated at least three times. Data from individual experiments are shown as dot-blots. Data are described as x-fold change compared to unstimulated control or percentage of remaining inflammation compared to stimulated controls. Stimulated controls are cells exposed to either LPS, saliva, or IL-1β and TNFα that initiated an inflammatory response. Statistical analyses were based on Pair t-test, Kruskal-Wallis, and Mann-Whitney test and an ANOVA. Data were analyzed by the Prism 7.0e software (GraphPad Software; San Diego, CA, USA).

## Figures and Tables

**Table 1:** Secretome of irradiated PBMCs suppresses inflammation in Nrf2 knockout macrophages.

	LPS	LPS+Secretome	SLV	SLV+Secretome
Nrf2 KO (1)	149.6	93.5	539.4	182.9
Nrf2 KO (2)	273.8	251.9	297.5	234.2
WT (1)	458.0	88.6	510.4	76.1
WT (2)	342.o	92.1	404.0	118.2

Murine bone marrow-derived macrophages from Nrf2 knockout mice and the respective controls (WT) were exposed to the secretome corresponding to 1 × 10^7^ irradiated PBMCs/mL. The inflammation was provoked by 100 ng/mL LPS or 5% saliva (SLV). The data show the x-fold increase of IL1 expression compared to unstimulated control. Data show two independent experiments (1, 2).

**Table 2:** The primer sequences.

Primers.	Sequence_F	Sequence_R
hIL1	CTGATGGCCCTAAACAGATGAAGT	AGCCCTTGCTGTAG TGGTGGT
hIL6	GAAAGGAGACATGTAACAAGAGT	GATTTTCACCAGGCAAGTCT
hIL8	AACTTCTCCACAACCCTCTG	TTGGCAGC CTTCCTGATTTC
hGAPDH	AAGCCACATCGCTC AGACAC	GCCCAATACGACCAAATCC
hACTIN	CCAACCGCGAGAAGATGA	CCAGAGGCGTACAGGGATAG
mIL1β	AAGGGCTGCTTCCAAACCTTTGAC	ATACTGCCTGCCTGAAGCTCTTGT
mIL6	GCTACCAAACTGGATATAATCAGGA	CCAGGTAGCTATGGTACTCCAGAA
mGAPDH	AACTTTGGCATTGTCGAACG	GGATGCAGGGATGATGTTCT
mACTIN	CTAAGGCCAACCGTGAAAAG	ACCAGAGGCATACAGGGACA

## Supplementary Materials

Supplementary materials can be found at https://www.mdpi.com/1422-0067/21/13/4694/s1.
