# Supplementary material for: Role for Lipids Secreted by Irradiated Peripheral Blood Mononuclear Cells in Inflammatory Resolution in Vitro
Source: Int J Mol Sci. 2020 Jun 30;21(13):4694. doi: 10.3390/ijms21134694 (PMC7370068; doi:10.3390/ijms21134694)
Supplement: Supplementary file 1 [file ijms-21-04694-s001.pdf]

International Journal of
Molecular Sciences

MDPI

Supplement Figures

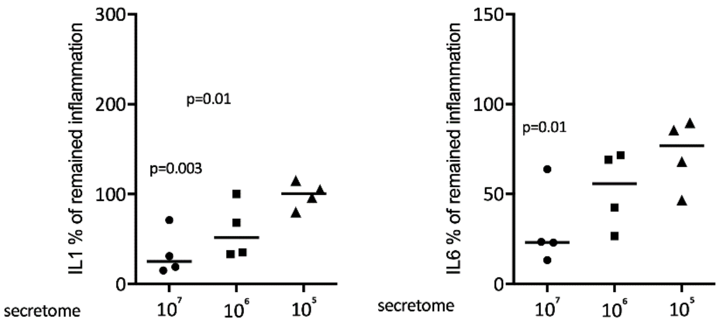

Figure S1. Secretome of irradiated PBMCs suppresses inflammation in primary macrophages. Murine bone marrow-derived macrophages were exposed to secretome at concentration equivalent to 10^7 to 10^5 PBMCs/ml. Inflammation was provoked by LPS 100 ng/ml Expression of inflammatory genes is indicated in percentage (%) compared to stimulated controls (100%). Dot-blots represent independent experiments. P-values are based on an ANOVA with multiple comparison, related to secretome 10^5 PBMCs/ml.
